# Laser-Assisted Periodontal Management of Drug-Induced Gingival Overgrowth under General Anesthesia: A Viable Option

**DOI:** 10.1155/2013/387453

**Published:** 2013-05-29

**Authors:** Tupili Muralikrishna, Butchibabu Kalakonda, Sumanth Gunupati, Pradeep Koppolu

**Affiliations:** ^1^Department of Periodontics, Sri Sai College of Dental Surgery, Kothrepally, Vikarabad, Andhra Pradesh 501101, India; ^2^Department of Periodontics, Narayana Dental College and Hospital, Chinthareddypalem, Nellore, Andhra Pradesh 524003, India

## Abstract

Gingival overgrowth/hyperplasia can be attributed to several causes, but drug-induced gingival overgrowth/hyperplasia arises secondarily to prolonged use of antihypertensive drugs, anticonvulsants and immunosuppressants. The management is complex in nature considering the multitude of factors involved such as substitution of drug strict plaque control along with excision of the tissue to be performed under local anesthesia as outpatient. In the recent times, the patient's psychological fear of the treatment with the use of surgical blade and multiple visits has developed the concept of single visit treatment under general anesthesia incorporating a laser as viable option. The present case highlights the new method of management of gingival overgrowth.

## 1. Introduction

Different types of periodontal therapy, both surgical and nonsurgical, have been attempted to either reduce or eliminate pockets associated with drug-induced gingival overgrowth (DIGO). Surgical treatment proved to be the decisive therapy of DIGO. Many of the surgical approaches such as gingivectomy and flap surgery are carried out under local anesthesia but certain cases warrant treatment under general anesthesia. The present case report illustrates management of DIGO under general anesthesia. The management consists of oral hygiene procedures, drug substitution, and a surgical gingivectomy.

## 2. Case Description

**Figure 1:**
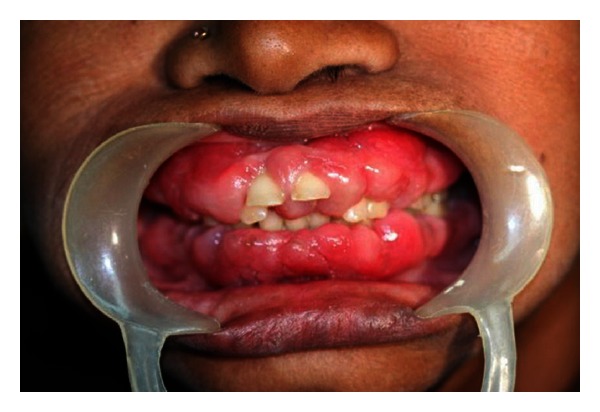
Intraoral photograph of amlodipine-induced gingival overgrowth.

A 55-year-old female patient presented to our hospital for evaluation of painless gingival overgrowth in both the upper and lower jaws which is posing her masticatory problems. Patient's medical history revealed that she was hypertensive and was on amlodipine 10 mg once daily for the past 4 years. Intraoral examination revealed generalized diffuse gingival enlargement, fibrotic in nature covering more than 2/3 rd of the crowns with minimal inflammation (Figure 1). 

Initial treatment consisting essentially of supragingival scaling was done, and the patient was advised on proper plaque control. The treating physician was consulted for a change in the medication regimen. The physician promptly obliged and put the patient on an alternate regimen of losartan. The review after one week revealed some reduction of the inflammatory component in the lower arch. The clinical diagnosis was suggestive of DIGO. To confirm the diagnosis by histopathological means, a laser-assisted biopsy was suggested. To allay patient's fear about needle and injections, a decision was made to remove the gingival overgrowth for biopsy with a laser. 

**Figure 2:**
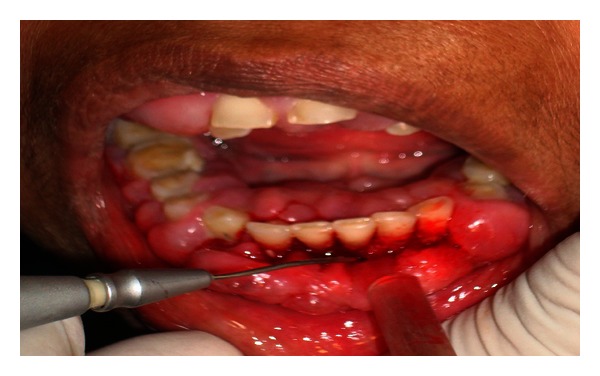
Excision of the enlargement for biopsy with a Nd:YAG laser.

**Figure 3:**
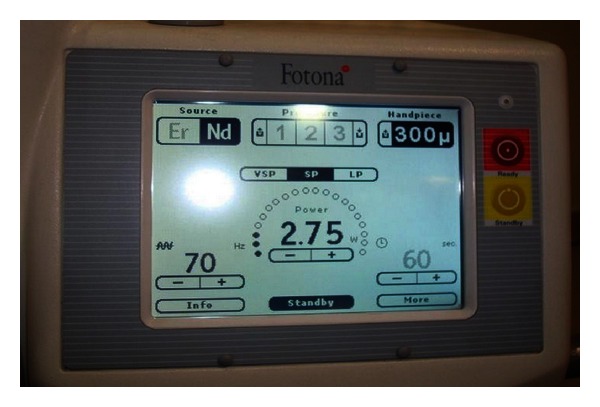
Laser settings for excision of the tissue.

**Figure 4:**
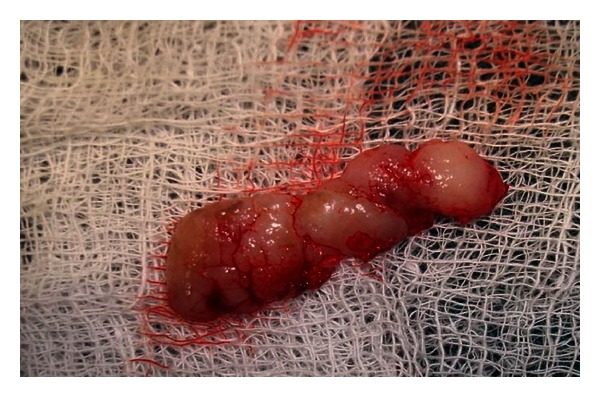
Excised specimen sent for histopathological examination.

After appropriate eye protection wear was used, a topical anesthetic gel was applied for 2-3 min. Nd:YAG laser (AT Fidelis, Fotona, Germany) was used with a 300 *μ*m fiber at 2.75 watts to dissect the overgrowth from its periphery (Figure 2). These laser settings were ideally chosen because peak power was required to penetrate the thick gingiva (Figure 3). The excised tissue was sent for histopathological examination (Figure 4). There was absolutely no bleeding; the patient was comfortable throughout the procedure and was happy that sutures were not necessary.

**Figure 5:**
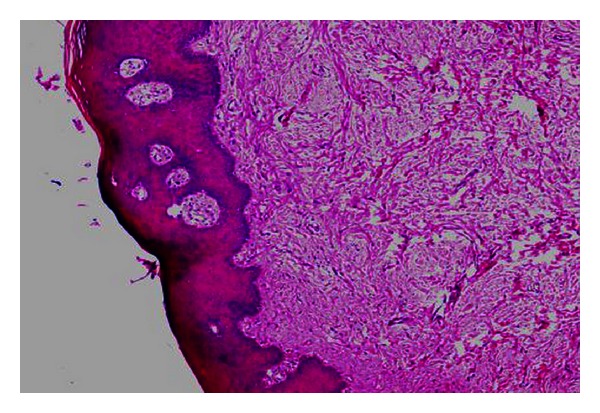
Histopathological view of the excised specimen shows stratified squamous epithelium with proliferating rete ridges; connective tissue showing abundant plump and proliferating fibroblasts that are spindle shaped forming a network. Few endothelial lined blood vessels are also observed (H&E, 10x).

The histopathological examination on staining the tissue with Hematoxylin and Eosin (H&E) showed hyperkeratotic stratified squamous epithelium with proliferating rete ridges, connective tissue showing abundant plump, and proliferating fibroblasts that are spindle shaped forming a network. Few endothelial lined blood vessels are also observed, suggestive of fibrous gingival hyperplasia (Figure 5).

Considering the cumbersome task of managing a massive gingival enlargement coupled with uncooperative and apprehensive nature of the patient, we decided to perform the surgery under general anesthesia. 

Fitness for surgery under general anesthesia was approved by the patient's physician and the anesthetist, and the patient was instructed to report to our hospital a day before the surgery. The patient was thoroughly informed about the surgical procedure, and consent was taken. Preanesthetic medication was given.

**Figure 6:**
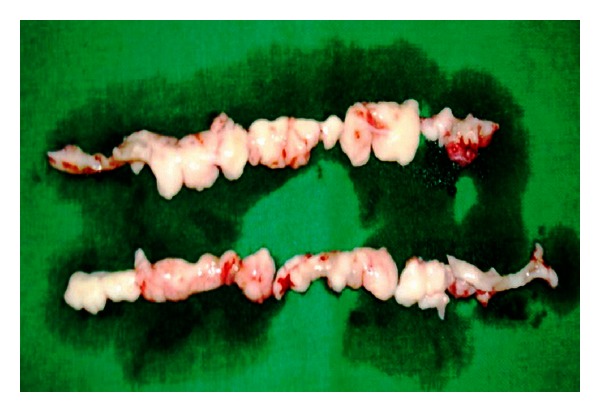
Excised tissue after gingivectomy.

Supplemental anesthesia, that is, local infiltration with 2% lidocaine with adrenaline (1 : 100000), was given to minimize bleeding during surgery. External bevel gingivectomy was carried out in both maxillary and mandibular arches using Bard Parker blades nos. 11 and 15. Excised tissue was removed using Gracey curettes and gingivectomy knives (Kirkland and Orban) (Figure 6). Laser was used to recontour the gingiva and aid in hemostasis.

**Figure 7:**
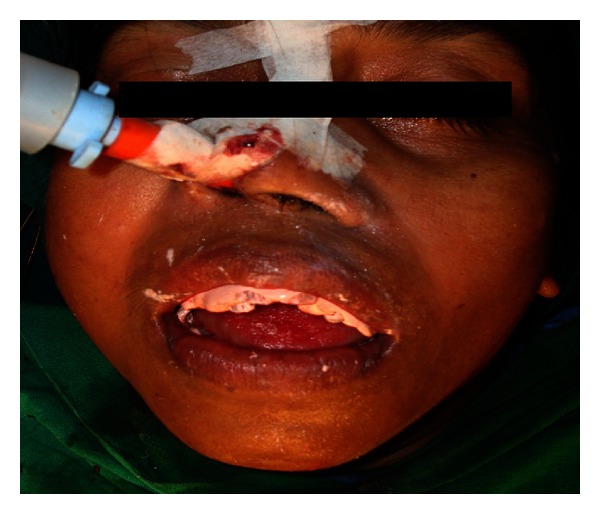
Patient at the end of the surgery and after the pack placement.

**Figure 8:**
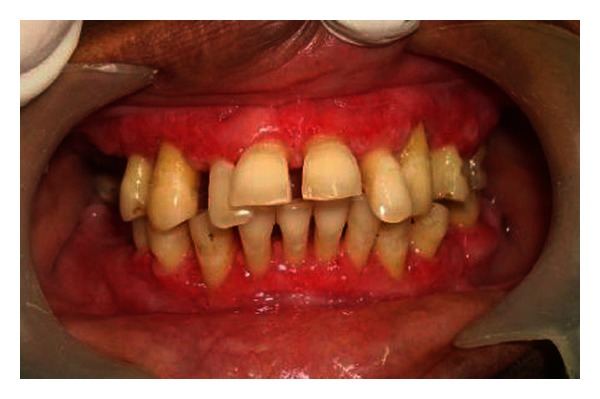
Postoperative view depicting uneventful healing.

After recovery from general anesthesia, a periodontal dressing Coe-Pack (GC International Inc., Newport Pagnell, UK) was given (Figure 7). The patient was kept under observation aftersurgery and was discharged 3 days after the procedure. There were no postoperative complications, and healing was uneventful (Figure 8). At the most recent followup, after the procedure, no recurrence of the hyperplasia was found.

## 3. Discussion

Fear, inability to cooperate, and trauma associated with injections are the most frequent indications which necessitate the option of general anesthesia. There are hardly few case reports where periodontal surgery was carried out under general anesthesia [1].

In our present case report, the patient underwent excision of her gingival overgrowth under local anesthesia previously and was unwilling to undergo the same procedure once again under local anesthesia as the last surgical procedure left the patient traumatized and made her apprehensive to undergo any further dental treatment. Hence periodontal management under general anesthesia was the viable option considering the massive gingival overgrowth, patient's apprehensive nature, and her medical status. 

Many studies emphasize the association between oral hygiene status and the severity of DIGO [2, 3]. Hence this clearly suggests that the degree or severity of gingival enlargement is commensurate with plaque-induced gingival inflammation. In the present case report also the patient's poor plaque control coupled with the massive gingival overgrowth made oral hygiene maintenance a nightmare. There was mild reduction in the inflammatory component of the gingival overgrowth in the upper arch after scaling. Hence the key to manage the DIGO lies in good oral hygiene and patient's compliance. 

Calcium channel blockers are known to cause gingival overgrowth. The prevalence of amlodipine-induced gingival overgrowth was 3.3%, half of nifedipine, that is, 6.3% [3]. As a first line of treatment for reduction of gingival overgrowth and anticipating recurrence after surgery, drug withdrawal or substitution was considered.

 Amlodipine was substituted to Losartan 50 mg once daily as per the patient's physician. Patient followup was done for 3 months to notice any advantage of change in medication that might have led to reduction in gingival overgrowth. But unfortunately only mild reduction of gingival overgrowth in the lower anterior teeth had prompted us to opt for a corrective surgery. Literature review and few case reports suggest reduction of gingival overgrowth within weeks to months after drug withdrawal [4, 5]. However, it is unfortunate that not many patients with longstanding gingival overgrowth respond to drug substitution alone [6, 7].

Performing periodontal surgery under general anesthesia involves some amount of risk. Careful selection of patients is mandatory to ensure that the treatment is successful. We have taken into consideration the patient selection criteria given by the American Society of Anesthesiologist (ASA) physical status classification [8]. According to the above-mentioned classification, the patient in this present case report was given an ASA class 2 category since she is a wellcontrolled hypertensive patient with blood pressure reading of 140/90 mm Hg. 

Preoperative screening was done and a detailed medical history was taken. Thorough evaluation of the patient's respiratory, renal, and cardiovascular systems was done with assessment of blood pressure. The treatment plan for this case is essentially comprised of an external bevel gingivectomy under general anesthesia supplemented by the use of laser to recontour the tissue and aid in hemostasis. 

Gingivectomy was considered as the first treatment of choice for DIGO [6]. Various other modalities of treatment for the management of DIGO comprise electrosurgery [9], laser gingivectomy [10], and flap surgery [11]. Considering the massive gingival overgrowth and the operational difficulties such as suturing associated with flap surgery, we considered external bevel gingivectomy as the final option. Also taking into consideration the esthetic outcomes, comparing both flap surgery and scalpel gingivectomy, those treated by the latter method appeared to have a “smoother” gingival surface than when treated with flap procedure [12].

The recovery of the patient after surgery was uneventful. Patient was evaluated for any post surgical bleeding. There are a few studies on recurrence rates after a scalpel gingivectomy but in this present case report, patient followup after 3 months showed no signs of recurrence.

## 4. Conclusion

Periodontal surgery under general anesthesia offers an attractive alternative for patients who are apprehensive or traumatized by earlier dental treatments. General anesthesia could be the preferred option in managing cases with massive gingival enlargements which would otherwise require multiple visits under local anesthesia. But it is imperative for the periodontist to have a sound knowledge before recommending patients to undergo surgical procedures under general anesthesia.
